# Microstructure and Mechanical Properties of Low-Cost SiC-Reinforced Aluminum and Al4Cu Matrix Composites Produced by Sintering in Vacuum

**DOI:** 10.3390/ma16155492

**Published:** 2023-08-07

**Authors:** Anna Wąsik, Beata Leszczyńska-Madej, Marcin Madej, Marcin Goły

**Affiliations:** 1 Faculty of Non-Ferrous Metals, AGH University of Science and Technology, 30 Mickiewicza Ave., 30-059 Krakow, Poland; anna.wasik@agh.edu.pl; 2 Faculty of Metals Engineering and Industrial Computer Science, AGH University of Science and Technology, 30 Mickiewicza Ave., 30-059 Krakow, Poland; mmadej@agh.edu.pl (M.M.); marcing@agh.edu.pl (M.G.)

**Keywords:** aluminum matrix composites, powder metallurgy, vacuum sintering, microstructure, mechanical properties

## Abstract

Composite materials based on Al and Al4Cu with the addition of SiC particles (2.5; 5; 7.5; 10 wt.%) were produced in low-cost conventional powder metallurgy processes involving mixing, compacting with a pressure of 300 MPa, and sintering at 600 °C in a vacuum atmosphere. An attempt was made to create a relationship between the vacuum sintering and the microstructure and mechanical properties of Al/SiC composites. The strength of the matrix-reinforcing interface depends on the chemical composition of the components; therefore, the influence of 4 wt.% copper in the aluminum matrix was investigated. Comprehensive microstructural and mechanical properties (including Brinell hardness, compressive and flexural strength measurements) of the produced composites were measured. The addition of 2.5 wt.% SiC to the Al4Cu matrix improved the mechanical properties of the composites compared to the matrix. In the composite with the addition of 2.5 wt.% of SiC, while the addition of the reinforcement did not affect the hardness and compressive strength and caused a rapid decrease in the flexural strength compared to the aluminum matrix, the addition of Cu to the matrix of this composite improved hardness (from 25 to 49 HB), compressive strength (from 423 to 618 MPa), and flexural strength (from 52 to 355 MPa).

## 1. Introduction

Striving to reduce the weight of elements while maintaining high operational and utility properties that meet economic requirements at the same time is the main motivation inspiring the rapid development of composite materials. An indispensable criterion in the technological process is the low cost of the product. Cheap and environmentally neutral solutions are constantly sought. Powder metallurgy is the most commonly applied method in manufacturing metal matrix composites because of the possibility of transformation of any powder into a finished product. It has found application in the automotive, defense, and aerospace industries [1]. The solid-state technology route of powder metallurgy is an energy-saving process that allows the easy and economical formation of composite sinters of various compositions by simple mixing, pressing, and free sintering of powder mixtures, characterized by better quality and low price [2,3]. Free sintering, i.e., without pressure, is used in the formation of powders sintered in a furnace without pressure. It is the most common and economical sintering method. In addition, the cost reduction of the production process is influenced by the possibility of using a relatively low sintering temperature to produce small parts based on aluminum powder. Furthermore, the use of ready-made die parts of the appropriate size and shape minimizes the need for machining the product and the associated material waste [4,5]. Powder metallurgy is also a very efficient method applied in the recycling of metals and alloys [6]. It should also be noted that the process utilizes temperatures below the melting point of the treated material [7]. Therefore, in the process of fabricating Al/SiC composites using the powder metallurgy method, due to the low sintering temperature, the interfacial reactions between Al and SiC that cause the formation of the brittle Al_4_C_3_ phase, which can degrade mechanical properties, are limited [8]. SiC-reinforced aluminum matrix composites, due to their desirable mechanical and physical properties, have attracted the attention of the aerospace, automobile, and electronic packaging industries [9,10]. SiC particles are characterized by a combination of low density and high hardness, elastic modulus, and superb thermal conductivity [11,12]. Additionally, it is said to be a cheap carbide. The addition of SiC particles, as a reinforced phase, to the aluminum matrix improves strength, specific modulus, and wear resistance [13,14]. Jianian Hu et al. [15] observed a remarkable strength effect of Al/SiC composites compared to pure Al. An improvement in the mechanical properties of Al/SiC composites in relation to the aluminum alloy matrix was also achieved by Xiong et al. [16]. Xinghua Ji et al. [17] fabricated AlSi10Mg/SiC composites using a powder metallurgy method. The authors observed a 21% increase in tensile strength in the composite with the addition of 5 wt.% SiC compared to the matrix material. The increase in hardness and impact strength with the increase in SiC weight content in AA6061/SiC composites produced using powder metallurgy was observed by Mulugundam Siva Surya et al. [18]. The strengthening effects in composite materials result from the transfer of external loads from the softened matrix to the hard ceramic reinforcement during the deformation process but also from the variability in the nature of the matrix microstructure, which is influenced by many mechanisms resulting from the addition of the reinforcing phase [19]. Therefore, the final properties of the composite, apart from the amount and size of the reinforcing phase and its homogeneous distribution in the matrix, will also be affected by the type of material used in the matrix of the composite. The effective transfer of external stresses is influenced by the interfacial adhesion between the matrix and the reinforcement phase. The Al/SiC interfacial character might be changed by alloying the aluminum matrix with different elemental powders to form the liquid phase during the sintering process. Modification of the matrix/reinforcement interface occurs through the segregation of an alloying element from the interface and its reaction with the reinforcement phase [20]. The most common alloying elements of aluminum powder are Cu, Mg, Zn, etc. Due to the possibility of additional age hardening of Al-Cu alloys, copper is the predominant alloying element for aluminum. During the Al-Cu sintering process, the liquid forms at 548 °C as a eutectic on the Al-Al_2_Cu boundaries [21]. The presence of a liquid phase during the sintering process can improve the diffusion between powders as it penetrates the grain boundaries [22].

In this work, composite materials based on aluminum and Al4Cu alloy with the addition of SiC particles were produced in conventional powder metallurgy processes, which allows for a reduction in the cost of the technological process while maintaining good mechanical properties. The production process consisted of the following stages: mixing, pressing, and sintering in vacuum. The sintering process has a significant impact on the microstructure and final physical and mechanical properties of the produced materials. The phenomena and processes that occur during sintering have a decisive influence on the durability of the metal–ceramic interface. In the research conducted, an attempt was made to explain the influence of the vacuum atmosphere on the sintering process on the microstructure and mechanical properties of Al/SiC composites. Consequently, to assess the microstructure of the produced composite sinters, microstructural observations were carried out using various observation techniques. The strength of the matrix-reinforcing interface depends on the chemical composition of the components and the presence of oxides. Therefore, the influence of 4 wt.% copper in the aluminum matrix was investigated. The interaction between the components of the composite determines the adhesion of the matrix to the reinforcement surface and therefore has a direct impact on the mechanical properties of the composite. An attempt was made to determine the strength of the metal–ceramic systems through performing a hardness test, uniaxial compression, and a three-point bending test on the obtained materials and to develop a methodology to obtain a mechanically durable connection of the metallic matrix with ceramic particles of the reinforcing phase by modifying the chemical composition of the tested composites. The behavior of the Al-Cu diffusion couple in vacuum was also examined, especially in terms of overcoming the oxide layer on the surface of the Al particles. The results obtained for the microstructure and mechanical behavior of the examined materials contribute to the development of low-cost and high-performance composites with potential applications in engineering. Al/SiC composites are the answer to a number of applications in which the balance between the weight reduction of elements and maintaining high strength and mechanical properties is essential. The unique properties, which are provided by combining metal with ceramics, have allowed Al/SiC composites to find a number of applications in the automotive and aviation industries.

## 2. Materials and Methods

**Figure 1:**
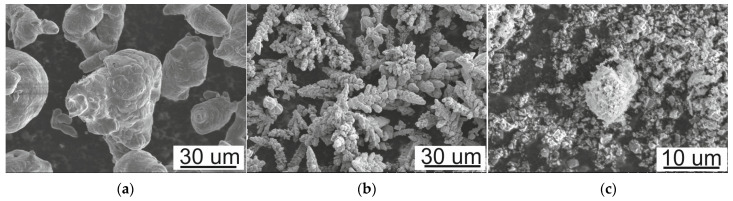
The morphology of the starting powders: (**a**) Al, (**b**) Cu, (**c**) SiC; SEM.

Commercially argon-atomized Al, electrolyzed Cu, and synthetized SiC powders were used as raw materials. Scanning electron microscope micrographs of the morphology of the starting powder materials are shown in Figure 1. According to the information provided by the manufacturer, the size of the irregular aluminum powder was below 63 µm (70% within the size 32–63 μm and the remaining <32 μm) with 99.7% purity. The size of the dendritic copper powder was 40 µm. The reinforcement phase in the form of SiC powder particulate was graded below 2 µm. The Al and Cu powders used as matrix material were mixed in a Turbula T2F (WAB, Muttenz, Switzerland) shaker mixer for 30 min. SiC particles were introduced in different weight fractions of 2.5 wt.%, 5 wt.%, 7.5 wt.%, and 10 wt.% in the matrix with a chemical composition of pure Al and Al with the addition of 4 wt.% of Cu. After mixing, the powder mixtures were compacted using a single-action compaction press with a pressure of 300 MPa into cuboids shapes with dimensions equal to 4.5 × 5 × 40 mm, without the addition of lubricants. The green bodies were subjected to a sintering process at 600 °C in a low vacuum of 10^−2^ Pa for 1 h. The sinters were slowly cooled with a furnace cooler. Following sintering, the composites were repressed and re-sintered for better consolidation, applying the same compaction and sintering parameters.

Subsequently, the density of the matrix materials (Al and Al4Cu), Al/SiC, and Al4Cu/SiC composites was determined using the Archimedes principle (ASTM-B962-17 [23]) with an accuracy of ±0.0001 g. The density was compared with the theoretical density, determined from the rule of mixtures, to estimate the volume of porosity in the materials. The microstructure of the fabricated composites was investigated with an Olympus GX 51 light microscope (LM) (Olympus, Tokyo, Japan), a Hitachi SU-70 Schottky-type electron gun scanning electron microscope (SEM) (Hitachi, Tokyo, Japan) with a Thermo Scientific NORAN System 7 X-ray microanalysis system (EDS) (Thermo Fisher Scientific, Waltham, MA, USA), and a JEOL JEM 2010 ARP transmission electron microscope (TEM) (Jeol Ltd., Tokyo, Japan). The phase composition was analyzed using an D8 Advance X-ray diffractometer (Bruker, Karlsruhe, Germany) with Co Kα = 1.79 Å. Comprehensive tests on the mechanical properties of the obtained materials were performed that included (i) hardness measurement using the Brinell hardness machine with a force of 31.25 kG, applying the 2.5 mm diameter carbide ball according to the ASTM E10-18 standard [24]; (ii) the determination of compressive strength according to the ASTM E9-89a standard [25] in a compression test performed at room temperature on a Zwick Roell Z050 (Zwick Roell, Ulm, Germany) universal test machine with an initial strain rate of 8 × 10^−3^ s^−1^ on specimens with a length/diameter ratio of 1.5; and (iii) the determination of bending strength in a three-point bending test (ASTM E290-22 [26]) carried out on the Zwick Roell Z050 test machine with a constant cross-head velocity equal to 0.05 mm/s and the length of the support span equal to 28 mm. Measurements of density, hardness, and flexural and compressive strength were carried out in three tests for each composition variant. In the hardness measurements, in each of the three tests, six indentations were made. The presented results are average values.

## 3. Results and Discussion

**Figure 2:**
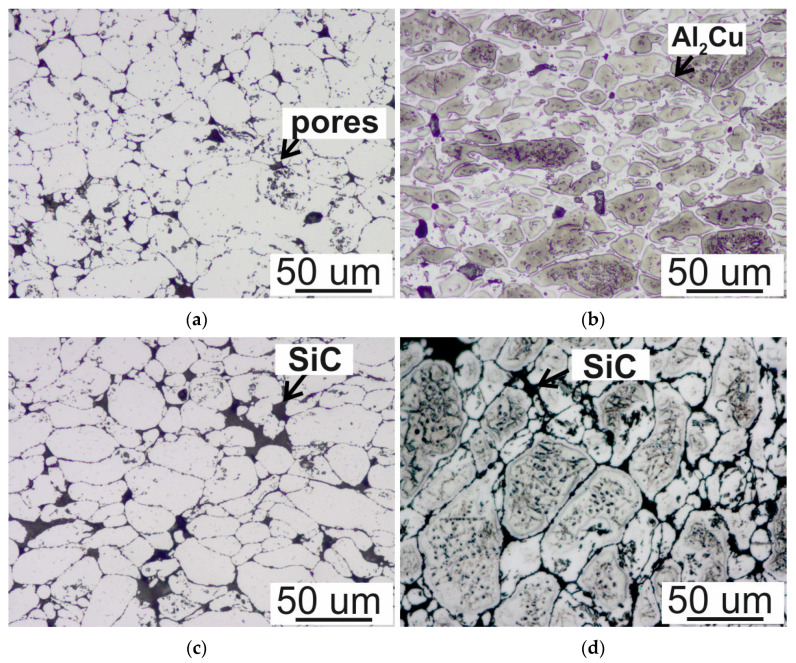
Optical microscopy images of (**a**) Al matrix, (**b**) Al4Cu matrix, (**c**) Al/SiC of 7.5 wt.%, (**d**) Al4Cu/SiC of 7.5 wt.%.

The optical micrographs of the sintered matrix materials and Al/SiC and Al4Cu/SiC composites with the addition of 7.5 wt.% of SiC are shown in Figure 2.

The microstructure of both the aluminum matrix and the Al4Cu alloy consists of regular grains. Additionally, in the Al4Cu alloy, numerous precipitations are visible at the grain boundaries and inside the grains. After slowly cooling, the morphology of precipitates exhibited the mixed character of linear (at the grain boundaries) and dotlike (inside the grains). At the same time, single pores are visible, also located at the grain boundaries. In composites with the addition of reinforcement, the SiC particles are located at the grain boundaries. Local agglomerates of the SiC phase are formed in the microstructure. A factor favoring the formation of agglomerations of the reinforcing phase is the relatively large ratio of matrix/reinforcement particle sizes [19] and the natural tendency to agglomerate fine powders, already during transport and storage.

**Figure 3:**
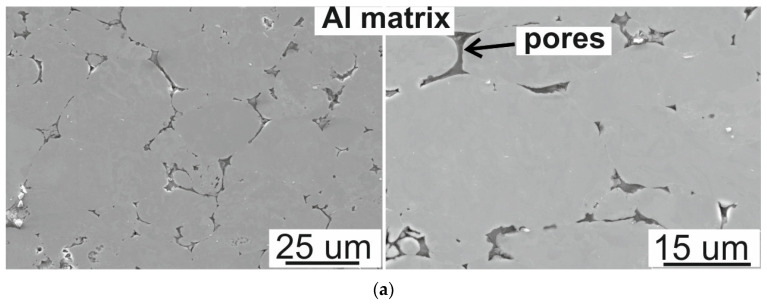

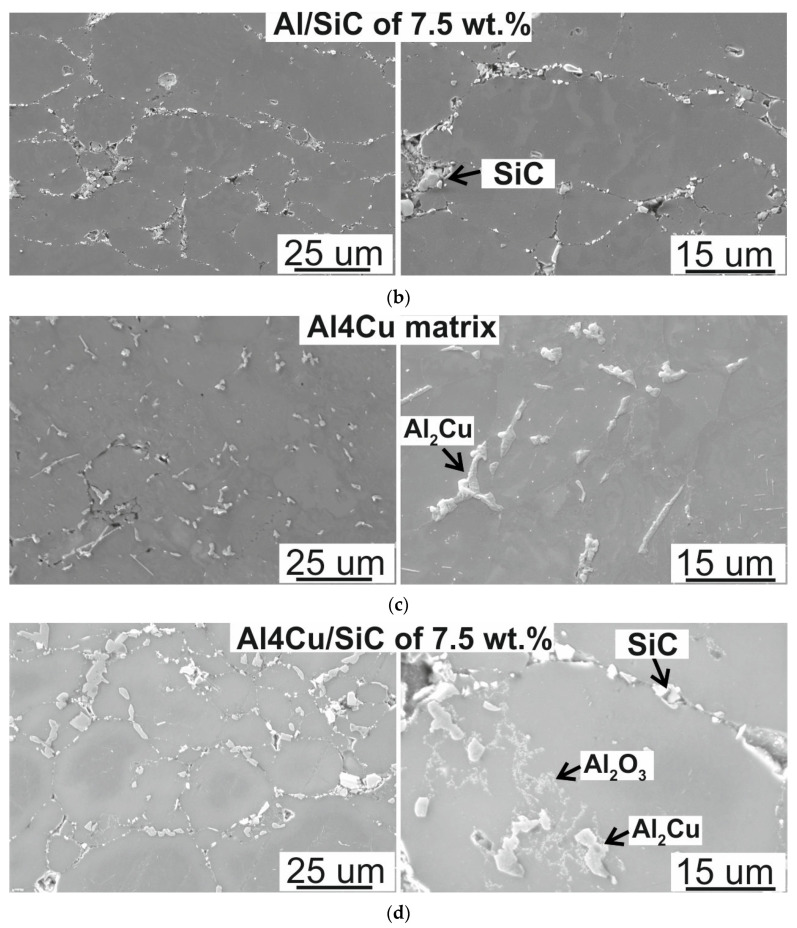
SEM images of (**a**) Al matrix, (**b**) Al/SiC of 7.5 wt.%, (**c**) Al4Cu matrix, (**d**) Al4Cu/SiC of 7.5 wt.%.

SEM micrographs (Figure 3) of Al-based composites show aluminum grains with a shape similar to spherical with pores at grain boundaries and voids between SiC particles, indicating poor powder consolidation resulting from the incomplete diffusion connection of powder particles in the sintering process. The copper-alloyed Al matrix composites show a better compacted microstructure. During the sintering process of Al-Cu, a series of Al-Cu intermetallic compounds are formed. Better densification of Al4Cu/SiC composites can be related to the presence of a liquid phase formed as a eutectic between aluminum and Al_2_Cu during sintering which by a capillary force filled most of the inter-particle boundaries, pores, and voids associated with the SiC agglomerations [22]. The sintering of the Al-Cu system at a temperature above the solidus temperature leads to the formation of a eutectic liquid according to reaction (1) [27]:

(1) $$\left(Al\right)+\theta \left({Al}_{2}Cu\right)↔L$$

**Figure 4:**
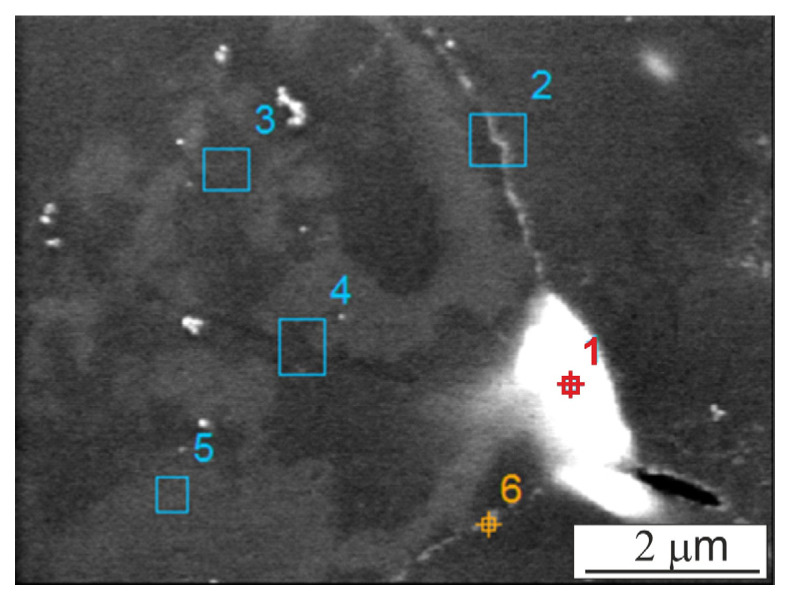
SEM micrograph of Al4Cu matrix.

The liquid migrates along the particle boundary; therefore, the microstructure of the Al4Cu matrix material consists of aluminum grains surrounded by coarse precipitates formed during slowly cooling with a furnace, which in SEM images are visible in the form of bright areas at the grain boundaries of aluminum grains (Figure 3). According to the EDS point analysis, shown in Figure 4 and Table 1, these precipitates are copper-rich phases with a copper content of 25% (point 1). From the same analysis, it can be seen that the aluminum matrix contains 3% copper (points 3 and 5). The above indicates that the copper is completely dissolved and absorbed into the aluminum matrix; no copper or other intermetallic compounds were observed. During the sintering process, and then as a result of slow cooling with the furnace, the copper precipitated in the form of the Al_2_Cu phase, although the matrix retained a small portion of copper. The formed liquid has the character of a vanishing liquid phase and there is too little of it to remove the pores located at the boundaries of the powder particles. An additional impediment to the action of the liquid phase are the non-wettable Al_2_O_3_ oxides present on the surface of the aluminum powder particles. From the point of view of the presence of the liquid phase, the use of vacuum as an atmosphere has a beneficial effect on the rate of liquid movement due to the removal of gases present in the open pores.

**Figure 5:**
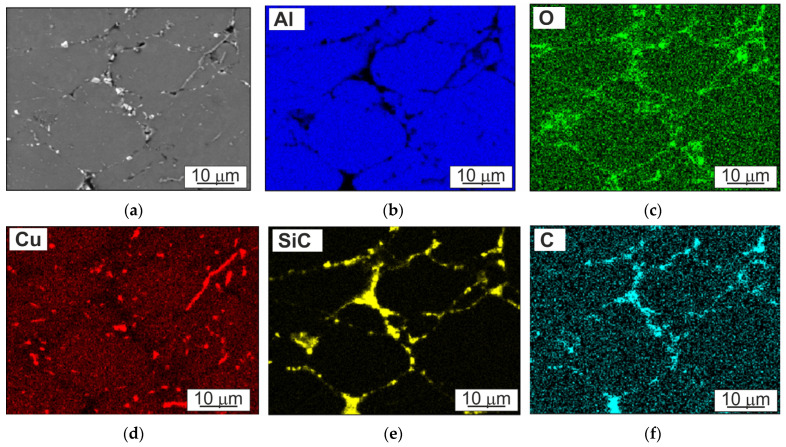
(**a**) SEM micrograph of Al4Cu/SiC of 10 wt.% and the corresponding EDS mapping micrographs of (**b**) Al, (**c**) O, (**d**) Cu, (**e**) SiC, (**f**) C.

**Figure 6:**
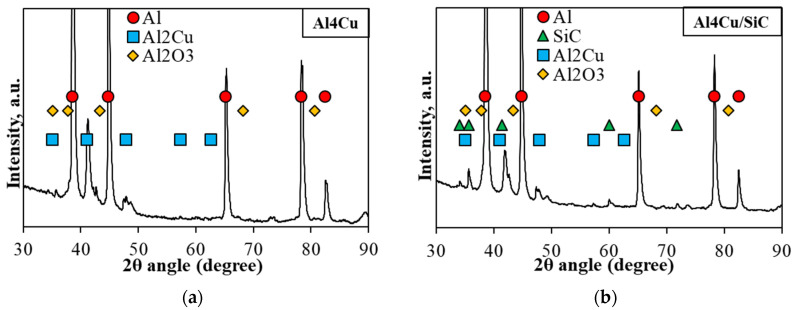
XRD plots of the (**a**) Al4Cu matrix and (**b**) Al4Cu/SiC of 5 wt.%.

The EDS map element distribution of the A4Cu matrix with the addition of 10 wt.% of SiC shown in Figure 5 illustrates the arrangement of the copper atoms which are dispersed on the particle boundary in the form of the second phase, and the rest remained within the grain. It additionally reveals the presence of oxides, which are located mainly at the grain boundaries, at the interface between aluminum and silicon carbide and in the pore areas as a network, which was also confirmed by X-ray diffraction (Figure 6). The surface of aluminum particles, due to its high affinity for oxygen, is covered with a layer of oxides, which are not reduced in the vacuum atmosphere used for sintering processes. Oxides present on the contact surface between powders can form a continuous barrier, isolating the direct contact of clean metallic surfaces. The XRD plots of the Al4Cu matrix and composite based on Al4Cu alloy with the addition of 5 wt.% of SiC also confirm that the sintered composite contains aluminum, Al_2_Cu precipitations, and the SiC phase in the composite material.

The presence of dissolved Cu in aluminum (Figure 4) and the Al_2_Cu precipitates present both at grain boundaries and residually within the aluminum grains indicate that the diffusion of copper into the Al matrix occurs despite the presence of an oxide layer on the surface of the Al powder. It can be assumed that this oxide layer is not continuous throughout the sintering process. One of the reasons for the violation of its cohesion may be the pressing process, where it undergoes local chipping. The loss of continuity of this oxide film on the surface of the original Al powder particles during sintering may be caused by the liquid phase moving under high pressure, since capillary forces are known to be dependent on the capillary size.

**Figure 7:**
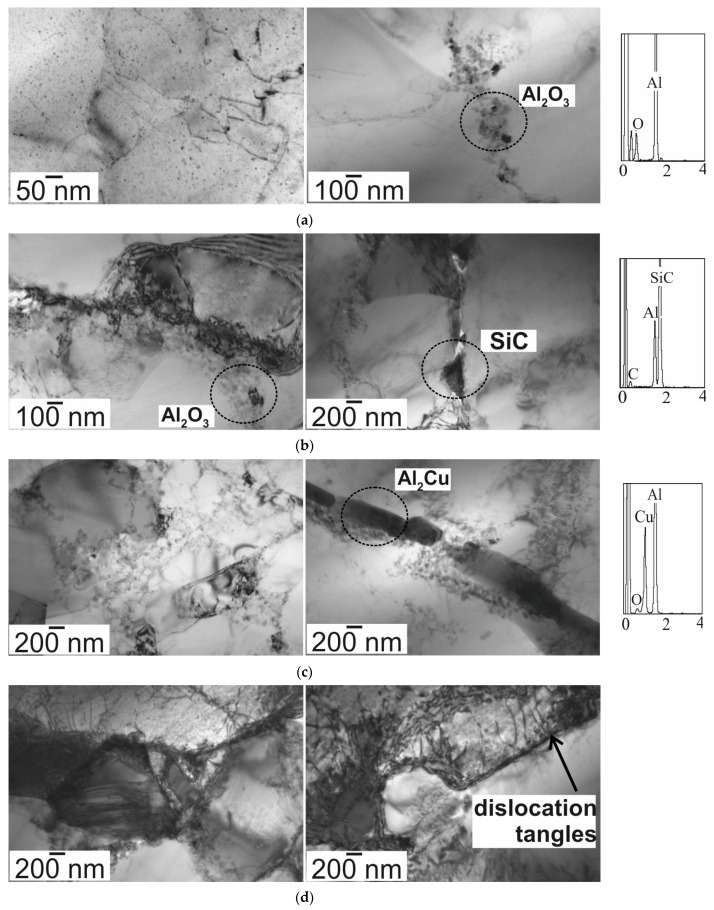
TEM images of (**a**) Al matrix, (**b**) Al/SiC of 5 wt.%, (**c**) Al4Cu matrix, (**d**) Al4Cu/SiC of 5 wt.%.

Figure 7 shows selected images of the microstructure of the tested materials, performed using transmission electron microscopy (TEM). The aluminum matrix consists of grains and subgrains of various sizes; additionally, the presence of fine, nanometric Al_2_O_3_ oxide accumulation and low-energy dislocation systems was found. In TEM images, the silicon carbide introduced into the aluminum matrix is visible as dark grey particles. In the aluminum matrix composite reinforced with 5 wt.% of SiC, an irregular transition zone with micro-pores and micro-cracks at the reinforcement–matrix interphase is visible, which indicated a weak bonding and adversely affected the mechanical properties. In the microstructure of the Al4Cu matrix, there are areas where dense dislocation entanglements have been revealed. A characteristic of the microstructure of these materials is the presence of fine precipitates of aluminum oxides, mainly located on the grain boundaries. The Al4Cu matrix and the composites based on it contain precipitates of the Al_2_Cu phase, visible in the TEM images in the form of dark elongated particles. In composites based on the Al4Cu alloy, the locally occurring subgrains are smaller than in composites based on aluminum. The size of grains and subgrains present in the microstructure, apart from SiC particles and Al_2_O_3_ oxides, is influenced by the addition of 4 wt.% Cu. The presence of dislocation loops was also characteristic, the number of which was much higher in the composites based on the Al4Cu alloy matrix. The implementation of hard silicon carbide ceramic particles in a plastic matrix due to the thermal expansion coefficient mismatch between the Al matrix (~23 × 10^−6^ K^−1^) and the SiC particles (~4.7 × 10^−6^ K^−1^) contributes to the generation of sufficient internal stresses for plastic deformation of the matrix, thus leading to the formation of a high density of dislocations at the interface of these two phases [15]. This difference in expansion coefficients induces local stresses, which can cause a loss of cohesion of the oxide film on the surface of the Al particles. Thus, if a liquid phase with copper is present, the diffusion of copper into the aluminum particles will be possible.

**Figure 8:**
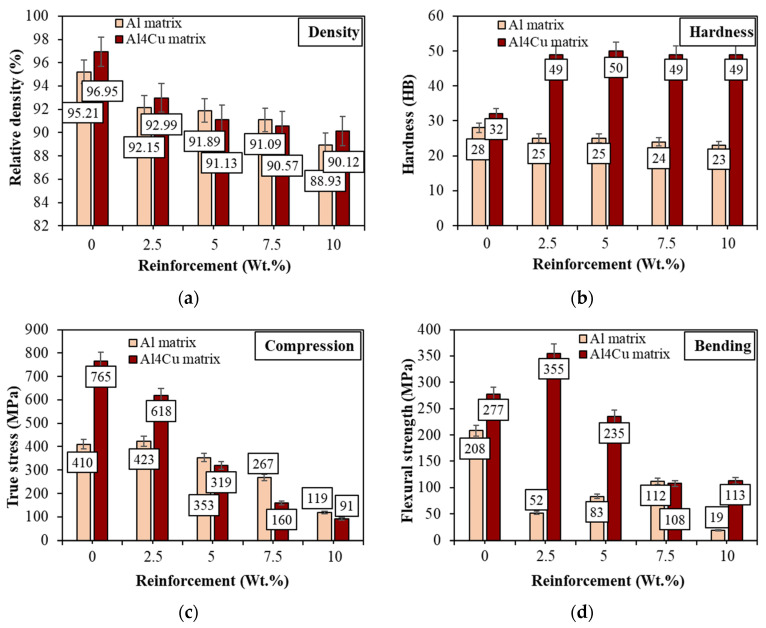
Variation of properties of (**a**) relative density, (**b**) Brinell hardness, (**c**) ultimate compressive strength, and (**d**) flexural strength of Al and Al4Cu matrix composites.

Figure 8 presents the variation in the physical and mechanical properties, including the density, hardness, compressive and flexural strength, of the obtained materials, depending on the chemical composition of the matrix and the weight fraction of the reinforcement phase. The density of the Al- and Al4Cu-based composites was measured using the Archimedes method (Figure 8a). The results show that the relative density for both aluminum and Al4Cu alloy matrix composites decreases with increasing weight content of SiC particles. At the same time, it can be observed that the addition of copper to the aluminum matrix allows for a higher densification of the materials. In contrast to the compaction and sintering of the single-phase materials, the addition of a hard, nondeformable reinforcing phase in a ductile matrix hinders the densification process and reduces the pressability of the material. Changes in relative density depending on the weight fraction of SiC are influenced by its tendency to form agglomerations in the voids between larger aluminum particles during the sintering process. Unfortunately, these agglomerates do not crumble when mixed in a Turbula-type mixer. Matrix densification is mainly facilitated by plastic deformation and grain boundary diffusion. However, in composites with the addition of a reinforcement phase, the presence of SiC particles hinders the diffusion across grain boundaries due to the “pinning of grain boundaries” by particles of the second phase, resulting in an increase in densification resistance [28]. The consolidation process of the material whose main component is aluminum is also affected by its high affinity for oxygen.

The surface of aluminum powder particles is already covered with a thin layer of oxides at the stage of production, which could block diffusion processes and make it difficult to obtain a high degree of compaction densification. The vacuum limits the oxidation of the aluminum powder during sintering, and the oxides are not reduced. However, as shown earlier, there are possibilities for pure metallic surfaces to appear even under these sintering conditions, especially in the presence of Cu and with the addition of SiC. Another persistent problem that affects the deterioration of the final properties of aluminum and its alloy matrix composites is the permanent oxide layer on the powder surface, formed as a result of self-passivation, and thus the problem of disrupting the initial continuous oxide layer in the sintering process. Hardness values (Figure 8b) indicate that while the addition of SiC particles to the copper-alloyed aluminum matrix allowed an increase in the hardness of the composites relative to the unreinforced material, in the case of Al/SiC composites, no increase in hardness of the material was observed as a result of the addition of SiC particles relative to pure aluminum; the hardness of both the matrix and composites with the addition of reinforcement oscillates at the level of 23–28 HB. However, in the Al4Cu/SiC composites, although the addition of SiC particles increased the hardness by approximately 17 HB relative to the Al4Cu matrix, the hardness values, regardless of the weight fraction of the SiC particles, remained at a constant level of approximately 49 HB. The addition of copper to the aluminum matrix allows higher hardness values to be achieved in the tested composites. While the difference in hardness between the aluminum matrix and the Al4Cu alloy is only 4 HB, in the case of the composite with the addition of 10 wt. % SiC, there is already a value of 26 HB. The increase in the hardness of composites in relation to the matrix is due to the addition of hard particles of the SiC strengthening phase and the formation at the metal–ceramic interface as a result of the difference in the thermal expansion coefficients of the matrix and the strengthening, dislocations in the form of dislocation loops that inhibit the movement of subsequent dislocations during plastic deformation, thus strengthening the material [29]. At the same time, it should be noted that the addition of 4% by weight copper to the aluminum matrix results in a twofold increase in hardness compared to aluminum matrix composites without the addition of copper. This confirms that copper is one of the most important aluminum alloying elements because of its high solubility and strengthening effect.

In Al/SiC and Al4Cu/SiC composites, strengthening with SiC particles caused a decrease in compressive strength, progressing with increasing reinforcement content (Figure 8c). The exception is the composite based on aluminum with the addition of 2.5 wt.% of the reinforcement phase, for which a higher compressive strength was observed compared to the pure matrix. Although for the Al4Cu aluminum alloy matrix, compressive stress values were almost twice as high as for the aluminum matrix, the introduction of the reinforcing phase resulted in their rapid decrease (from 618 MPa for 2.5 wt.% SiC to 91 MPa for 10 wt.% SiC). For Al/SiC composites, the increase in SiC content from 2.5 to 10 wt.% also resulted in a decrease in the stress values, but this decrease was not as rapid, and composites with the addition of 5 to 10 wt. % SiC in the aluminum matrix were characterized by a higher compressive strength compared to composites with the same strengthening content in the Al4Cu alloy.

Regardless of the chemical composition of the matrix material and the weight fraction of the reinforcing phase particles, the highest flexural strength is exhibited by the matrix of materials without the addition of reinforcement (Figure 8d). The exception is the composite based on Al4Cu aluminum alloy, in which the introduction of a small number of SiC particles (up to 2.5% by weight) caused an increase in bending strength relative to the matrix (from 277 MPa to 355 MPa). After this content was exceeded, there was a decrease in bending strength but less than that for composites based on aluminum. Lower values of flexural strength were characterized by Al-SiC composites. The difference in the values between aluminum sinter and reinforced composite by 2.5 wt.% SiC is 156 MPa. An increase in the weight fraction of the SiC phase to 10 wt.% caused a further decrease in flexural strength to 19 MPa. For comparison, the value of the flexural strength of an aluminum sinter is 208 MPa. For the composite with the addition of 7.5 wt.% SiC, the bending strength increased to 112 MPa, but it is almost twice as low as the pure matrix. The morphology (irregular shape) of the tested composites with the addition of SiC particles is responsible for the decrease in bending strength. The sharp edges of the SiC particles can promote crack formation during the bending test. Silicon carbide is a brittle phase; it also forms persistent clusters of fine particles, making it difficult to evenly distribute in the matrix during mixing in the Turbula mixer and also contributes to cracking. Similarly to the density and hardness of the composites, the addition of copper to the aluminum matrix resulted in a higher bending resistance of the composites, which proves their greater plasticity. The greatest influence of the addition of copper to the aluminum matrix on the results obtained in flexural strength is visible for the composite with the addition of 2.5 wt.% SiC and caused an increase in value by 303 MPa. It could be indicated that the liquid formed during the sintering of the Al-Cu system favorably influenced the improvement of the vacuum sintering quality, including an improvement in the cohesion between the sintered Al particles, which is likely to be related to the local crushing of the oxide layer as a result of the pressure associated with fluid movement.

**Figure 9:**
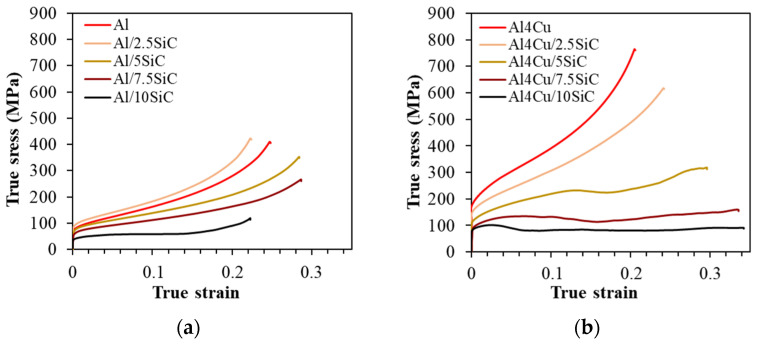
Compressive stress–strain curves of (**a**) Al matrix composites and (**b**) Al4Cu matrix composites.

**Figure 10:**
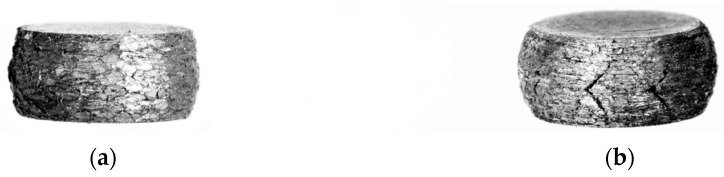
Exemplary photos of composite samples after compression of aluminum matrix composites with the addition of (**a**) 2.5 wt.% of SiC and (**b**) 7.5 wt.% of SiC.

The compressive stress–strain curves of the Al/SiC and Al4Cu/SiC composites show an increase in stress with an increase in strain (Figure 9). However, in composites with a higher content of the reinforcement phase (7.5 and 10% by weight), there is a continuous increase in the deformation of the material at a constant stress value equal to the yield point (material flow). The deterioration of mechanical properties due to the introduction of SiC phase particles may be caused by the concentration of internal stresses that result in interface cracking and reinforcing phase fracture. In addition, the introduction of a hard phase into the matrix hinders the densification process as a result of which the microstructure of the composites is characterized by a higher porosity, indicating insufficient consolidation of the powders compared to the matrix material. Furthermore, the agglomeration of the reinforcing phase contributes to the premature failure of composites and a reduction in their plasticity. The drop in yield strength caused by the addition of SiC particles to the Al and Al4Cu matrix can be explained by the secondary oxidation that may have occurred during low vacuum sintering, turning the coarse oxide film into a hard layer with low plasticity; therefore, the micro-deformation of the particle boundary is hindered by the hard and brittle oxide layer on the surface [27]. The photos of the samples after the compression test presented in Figure 10 indicate the occurrence of numerous cracks and chipping on the outer surface of the samples sintered in a vacuum atmosphere.

**Figure 11:**
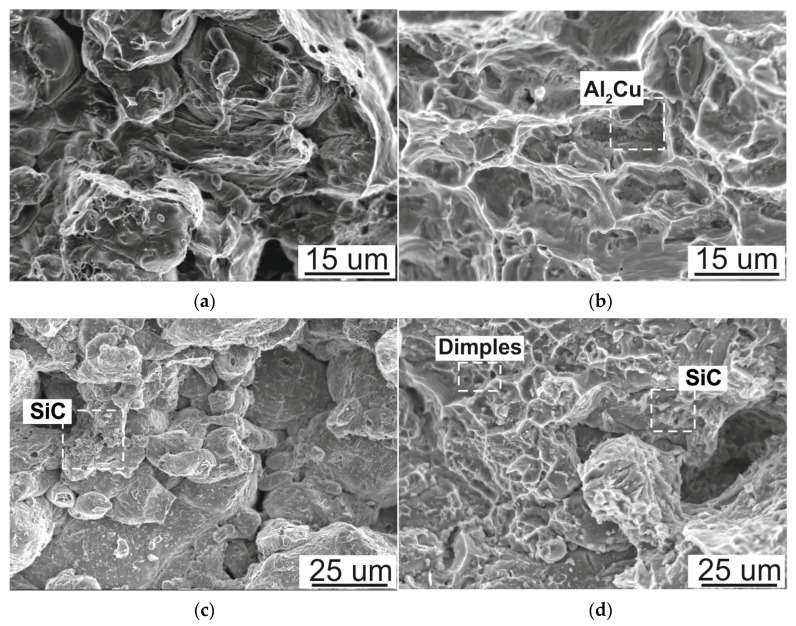
Fracture surfaces of (**a**) Al matrix, (**b**) Al4Cu matrix, (**c**) Al/SiC of 2.5 wt.%, (**d**) Al4Cu/SiC of 2.5 wt.%; SEM.

The fracture surfaces obtained after the three-point bending test were also observed using a scanning electron microscope (Figure 11). The fractures presented after the three-point bending test of Al and Al4Cu are ductile with visible elevations and dimples resulting from plastic deformation. Additionally, evenly distributed precipitates are visible on fractures based on the Al4Cu aluminum alloy. Locally, voids are also visible, which are probably pores. Fractures of the sinters indicate local insufficient connection between the particles, which causes them to easily decohere under the influence of the bending force. Fractures of composites with different contents of the SiC phase show a ductile character within both the aluminum and Al4Cu matrix, and brittle fracture occurs in the presence of SiC particles. In composite fractures, clusters of SiC particles are visible locally. With the presence of SiC particles, there is a loss of material cohesion; cracking is favored by clusters of SiC particles because of weak interface bonding and holes. The addition of copper into the aluminum matrix, due to the liquid formation, changed the character of the fracture surface, with dimples and tearing ridges, to a more plastic fracture. This confirms that the formation of a liquid phase improved the bonding quality.

## 4. Conclusions

The microstructure, mechanical properties, and effects of the addition of copper to aluminum matrix composite reinforced with various amounts of SiC particles prepared using low-cost powder metallurgy technology were investigated. The following conclusions can be drawn:

(1) The liquid phase formed in the Al4Cu matrix resulted in an increase in density during sintering and improved the mechanical properties. Numerous precipitations of Al_2_Cu are visible at the grain boundaries and within the grains.
(2) The fine SiC particles tended to agglomerate, hindering the densification. In composites, at the grain boundary and at the reinforcement–matrix interphase, fine pores forming a network were visible, which is a very unfavorable phenomenon that adversely affects the mechanical properties.
(3) According to the analysis, only the addition of a small amount of SiC (2.5 wt.%) into the Al4Cu matrix improved the mechanical properties of the composites relative to the matrix material. The deterioration of the mechanical properties has occurred as a result of the introduction of SiC phase particles into the aluminum matrix. The addition of copper resulted in a twofold increase in hardness and flexural strength compared to aluminum matrix composites.
(4) The deterioration of mechanical properties due to the introduction of SiC phase particles is associated with (i) the concentration of internal stresses due to matrix/reinforcement thermal expansion coefficient mismatch, (ii) a hindered densification process and limited pressability of composite materials due to the addition of a hard, nondeformable reinforcing phase in a ductile matrix, (iii) the agglomeration of the reinforcing phase and (iv) the barrier in the form of aluminum oxides, insulating the direct contact between the aluminum powders. The mechanical properties might be improved by applying pressure-assisted sintering as well as a higher-purity vacuum to avoid secondary oxidation.

## Figures and Tables

**Table 1:** EDS point analysis of Al4Cu matrix.

No	Al (wt.%)	Cu (wt.%)	O (wt.%)
1	74.06	24.61	1.33
2	97.48	0.90	1.62
3	97.04	2.96	-
4	96.53	2.93	0.55
5	96.36	2.95	0.70
6	92.54	5.70	1.76

## Data Availability

Not applicable.
